# Endotoxin Translocation and Gut Inflammation Are Increased in Broiler Chickens Receiving an Oral Lipopolysaccharide (LPS) Bolus during Heat Stress

**DOI:** 10.3390/toxins12100622

**Published:** 2020-09-29

**Authors:** Nicole Reisinger, Caroline Emsenhuber, Barbara Doupovec, Elisabeth Mayer, Gerd Schatzmayr, Veronika Nagl, Bertrand Grenier

**Affiliations:** BIOMIN Research Center, Technopark 1, 3430 Tulln an der Donau, Austria; caroline.emsenhuber@biomin.net (C.E.); barbara.doupovec@biomin.net (B.D.); e.mayer@biomin.net (E.M.); gerd.schatzmayr@biomin.net (G.S.); veronika.nagl@biomin.net (V.N.); bertrand.grenier@biomin.net (B.G.)

**Keywords:** endotoxins, lipopolysaccharides, broilers, heat stress, macrophages

## Abstract

Lipopolysaccharides (LPS), also termed endotoxins, are the major component of the outer membrane of Gram-negative bacteria. In general, endotoxins in the intestine are considered harmless in healthy animals. However, different stressors, such as heat stress, can lead to a compromised gut barrier, resulting in endotoxin translocation. Chickens are considered to be less sensitive to the effects of LPS compared with other species, for example, humans, pigs, or calves, probably because of the lack of the functional-specific TRAM-TRIF signalling pathway (MyD88-independent). Therefore, six LPS preparations (three different strains with two different preparation methods each) were compared in murine macrophages and characterized according to their MyD88-dependent pathway activation. All tested LPS preparations induced a strong inflammatory response after 4 and 24 h on a murine macrophage cell line. However, there was a similar strong response in the gene expression profile as well as production of nitrite oxide and TNF-alpha from LPS of different strains and preparation methods. On the basis of the results of the in vitro study, one LPS preparation was chosen for the subsequent in vivo study with broilers to assess the effect of an oral LPS bolus (*E. coli* O55:B5 phenol extracted; 2 mg/kg b.w.) during heat stress conditions (10 h, 36 °C). The most pronounced effects were seen in broilers receiving the oral LPS bolus during heat stress conditions. The endotoxin activity in the intestine as well as the serum concentration of the 3-OH C14 (part of LPS) were increased. In addition, an increased expression of genes related to inflammation and stress response (e.g., IL-6, IL-1beta, HSP70) was observed, whereas the expression of genes associated with gut health (e.g., MUC2, FABP2) was decreased. To conclude, an increase of intestinal LPS combined with heat stress can pose a risk to animal health.

## 1. Introduction

Lipopolysaccharides (LPS), also termed endotoxins, are the major component of the outer leaflet of the outer membrane of Gram-negative bacteria [1]. LPS cover approximately 75% of the cell surface [2] and are released from bacterial cells during cell division, cell death, or as a result of antibiotic treatment. It has been suggested that one bacterial cell, for example *Escherichia coli* (*E. coli*), contains several million LPS molecules [2]. The gastrointestinal tract hosts trillions of commensal bacteria, with both Gram-positive and Gram-negative bacteria. LPS are thus always present in the gut, whereas in healthy individuals, LPS are not absorbed into the circulation. Already in 1959, it was suggested that the intestine was the source of LPS found in the blood of patients with sepsis in a number of clinical conditions, as a result of gut barrier impairment [3].

LPS are composed of three different parts: the O-antigen, the core oligosaccharide, and the lipid A part. The latter is the most conserved and reactive part of the molecule. In addition to the bacterial source of LPS [4], preparation and purification of LPS have an influence on the inflammatory response in in vitro and in vivo experiments, as the amount of other components (e.g., proteins, RNA) in LPS preparations can vary. This variation can lead to a different response of the host or maybe even to differences in the mechanisms of signalling [5,6]. The response to intravenous (*i.v.*) administration of LPS is well studied and often used as a model for sepsis in humans [7]. Broilers seem to be relatively resistant to LPS compared with other species and the lethal dose is described to be above 50 mg/kg b.w. *i.v.* [8]. In comparison, the LD50 in mice is 10 mg/kg b.w. *i.v*., which is still high considering that 0.015 in humans and 0.025 mg LPS/kg b.w. *i.v.* in calves is required to induce a severe shock [9]. Resistance of broiler chickens to LPS could partly be explained by the toll-like receptor 4 (TLR4)/ myeloid differentiation factor 2 (MD2) complex, which has the unique property to lack the functional-specific TRIF-related adaptor molecule - TIR domain-containing adaptor protein inducing interferon beta (TRAM-TRIF) signalling pathway (MyD88-independent) [10]. Therefore, LPS may only activate the MyD88 dependent signalling route, implying the differential in vivo response in chicken compared with other species. Although LPS might not lead to mortality in chicken, *i.v.* administration can induce clinical signs (e.g., fever) and sickness behavior, and negatively impact performance [11,12,13]. Only a few studies have evaluated the effect of oral LPS administration on broiler health, using either LPS from *E. coli* or *Salmonella typhimurium* [14,15,16]. Repeated oral LPS administration of up to 0.5 mg/bird did not lead to reduced performance or clinical signs. In a study by Lucke et al. [17], oral LPS administration only affected genes related to mucosal gut barrier when LPS was combined with other stressors, such as the *Fusarium* mycotoxin deoxynivalenol.

Heat stress is another factor described to affect the gut barrier in broilers [18,19,20]. Annual loss in poultry industry due to heat stress is estimated to be about 128 to 165 million dollars [21]. Although animals have a certain capacity to adapt to heat stress, poultry seem to be especially sensitive to increased temperatures. Furthermore, modern poultry genotypes produce more heat as a result of their greater metabolic activity [22]. Several studies have already shown that heat stress has detrimental effects on broiler health, for example, altered behaviour and physiological homeostasis, decreased feed intake, increased resting time, increased panting and body temperature, or impaired reproduction function [23]. Furthermore, the gastrointestinal tract seems to be severely affected by heat stress. In rats and pigs, several studies are reporting a negative effect of heat stress on gut permeability [24,25] as well endotoxin translocation [25,26,27,28]. However, in broilers, there are only a limited number of studies assessing the effect of heat stress on gut permeability [19,29] or endotoxin translocation into the circulation [30]. In addition, no information on the quantity of endotoxins in the gut is available in healthy or challenged broilers. Intestinal endotoxin load can be influenced by pathogens as well as dietary challenges. These challenges might result in a drastic increase of the intestinal endotoxin load, as is already well described in ruminants during sub-acute ruminal acidosis [31]. Therefore, it is of interest to assess the endotoxin amount in the gut of broilers, and to examine whether oral endotoxin administration induces a local and/or systemic effect. Furthermore, animals are often exposed to more than one stressor. Thus, combining both oral LPS administration and heat stress might lead to new insights about the challenges occurring in broiler production.

The aims of our study were twofold. The first aim of the study was to identify which LPS preparations result in the strongest expression of the genes interleukin-1beta (IL-1beta), tumor necrosis factor alpha (TNF-alpha), and interleukin-6 (IL-6; as a result of MyD88-dependent pathway activation) and/or the lowest expression of interferon-beta (IFN-beta; type I IFNs, triggered via the MyD88-independent pathway) in a macrophage cell line. On the basis of these results, one LPS preparation was chosen for the subsequent in vivo study to investigate the dynamics of an oral LPS bolus in the gut, and to assess local and systemic effects of LPS combined with a single heat stress period in broilers.

## 2. Results

### 2.1. In Vitro Trials

Murine macrophages (RAW 264.7) were stimulated with LPS of two different *E. coli* strains (O55:B5, O111:B4) and one *Salmonella enteritica enteritidis* (*S. enteritica enteritidis*) strain either phenol extracted (PE) or phenol extracted and purified with ion-exchange chromatography (IC) for 4 h or 24 h to assess the expression of selected genes as well as the nitrite and TNF-alpha concentration in the supernatant. 

#### Effect of LPS Stimulation on Murine Macrophages after 4 and 24 H

**Figure 1:**
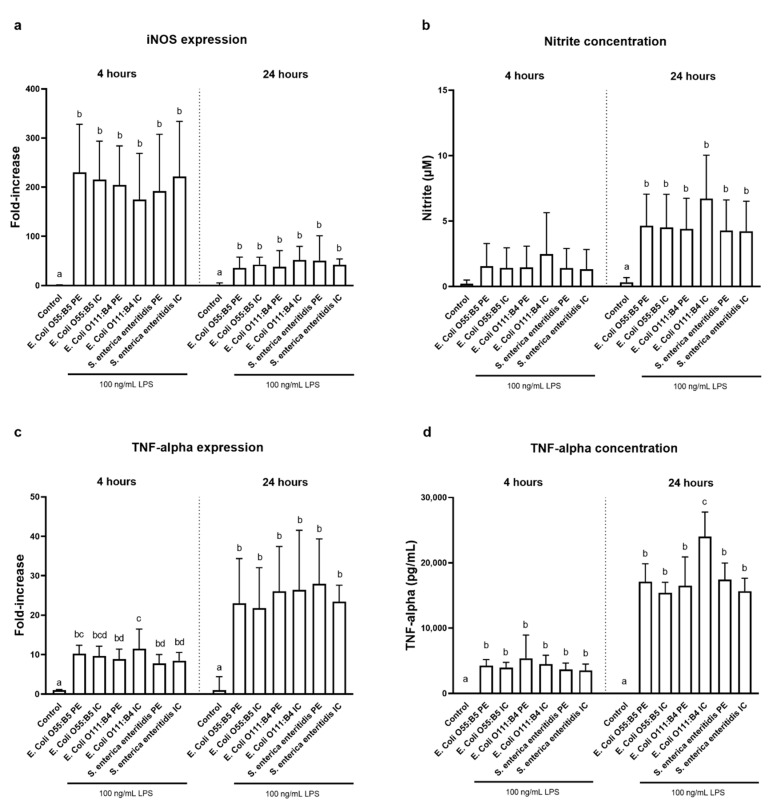
Gene expression of inducible nitric oxide synthase (iNOS) (**a**), nitrite concentration (µM) (**b**), gene expression of tumor necrosis factor alpha (TNF-alpha) (**c**), and TNF-alpha concentration (**d**) of RAW cells stimulated with 100 ng/mL lipopolysaccharides (LPS) of two different *Escherichia coli* (*E. coli*) strains (O55:B5, O111:B4) and one *Salmonella enteritica enteritidis* (*S. enteritica enteritidis)* strain either phenol extracted (PE) or phenol extracted and purified with ion-exchange chromatography (IC) for 4 h or 24 h (*n* = 6). Values represent mean values with standard deviation. ^abcd^ Superscripts indicate significant difference within treatment at each timepoint (*p* < 0.05).

All LPS preparations significantly increased the inducible nitric oxide synthase (iNOS) expression after 4 h (175- to 230-fold) and 24 h (36- to 52-fold) compared with the untreated control (Figure 1a). In contrast, nitrite concentration was only significantly increased after 24 h of LPS treatments (Figure 1b). There was no difference between LPS preparations for iNOS expression and nitrite concentration in the cell supernatant. Furthermore, all LPS treatments significantly increased the TNF-alpha expression after 4 h (8- to 10-fold) and 24 h (22- to 28-fold) compared with the untreated control (Figure 1c). Notably, cells stimulated with LPS from *E. coli* O111:B4 IC showed a significantly increased TNF-alpha expression after 4 h compared with LPS from *E. coli* O111:B4 PE and both LPS preparations from *S. enteritica enteritidis* (Figure 1c). Similar to the gene expression results, the TNF-alpha concentration was increased after 4 and 24 h (Figure 1d), respectively. Furthermore, LPS from *E. coli* O111:B4 IC significantly increased the TNF-alpha concentration at 24 h compared with all other LPS treatments (Figure 1d).

Yet, stimulation with any LPS preparation significantly affected the expression of selected genes after 4 and 24 h (Table 1 and Table 2). After 4 h of incubation, genes involved in TLR-signalling cascade as well as genes triggered following TLR activation were affected by LPS stimulation. Among genes involved in TLR signalling cascade, there was, interestingly, a significant reduction of the expression of TLR4, whereas expression of TLR2 was significantly increased. There was also a slight increase of the expression of CD14 and TICAM1 after 4 h LPS stimulation. The strongest increase was seen on the genes triggered following TLR activation (IL-1beta, TNF, IL-6, iNOS, and IFNB1) after 4 h LPS stimulation. Cells stimulated with LPS from *E. coli* O111:B4 IC showed a significantly increased IL-1beta expression after 4 h compared with LPS from *E. coli* O55:B5 IC and both LPS preparations from *S. enteritica enteritidis*. After 24 h stimulation, there was no effect on genes involved in TLR signalling cascade, except for a slight increase of TICAM1 with LPS from *E. coli* O55:B5 PE and *E. coli* O111:B4 IC. Only the expression of CD14 was slightly increased (twofold). The strongest effect was seen again on the genes triggered following TLR activation (IL-1beta, TNF-alpha, IL-6, and iNOS). Furthermore, the expression of IL-10 was significantly decreased by all LPS preparations. Incubation with *E. coli* O111:B4 IC led to a significantly higher fold-change of IL-1beta compared with all other LPS preparations. For all other genes, there was no significant difference in the expression regarding the LPS type or preparation method.

### 2.2. In Vivo Trial

An in vivo trial was conducted to investigate the local and systemic effects of an oral LPS bolus in broilers under heat stress conditions in broiler. Therefore, broilers were kept either under thermoneutral conditions (10 h, 23 °C) or under heat stress conditions (10 h, 36 °C) receiving an oral dose of saline (0.9%; no LPS) or LPS (1 × 2 mg/kg b.w.).

#### 2.2.1. Body Weight

There was no significant difference of body weight between groups on days 0, 14, and 29 (Supplementary Table S1).

#### 2.2.2. Endotoxin Activity in the Intestinal Digesta

**Figure 2:**
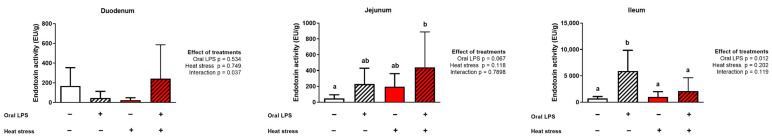
Endotoxin activity (EU/g) in the (**a**) duodenum, (**b**) jejunum, and (**c**) ileum of broilers kept either under thermoneutral conditions (10 h, 23 °C) or under heat stress conditions (10 h, 36 °C) receiving an oral dose of saline (0.9%; no LPS) or LPS (1 × 2 mg/kg b.w.). *n* = 8 animals/treatment. Values present means with standard deviation. − indicates no treatment, + indicates treatment. ^ab^ Superscripts indicate significant difference (*p* < 0.05).

Overall, endotoxin activity was significantly higher in the ileum compared with the duodenum and jejunum (*p* < 0.05). There was no effect of any treatment on the endotoxin activity in the duodenum (Figure 2a). In contrast, the endotoxin activity in jejunum was significantly increased (ninefold) in heat stressed animals receiving LPS compared with the thermoneutral group receiving no LPS (*p* < 0.05) (Figure 2b). In addition, there was a significant increase (eightfold) of the endotoxin activity in the ileum of thermoneutral animals receiving LPS compared with animals receiving no LPS (*p* < 0.05; Figure 2c). Overall, there was a significant effect of LPS administration on endotoxin activity in the ileum (*p* < 0.05) and a trend in the jejunum (*p* = 0.067).

#### 2.2.3. Gene Expression Analyses of the Intestine—Treatment and Interaction Analysis

**Figure 3:**
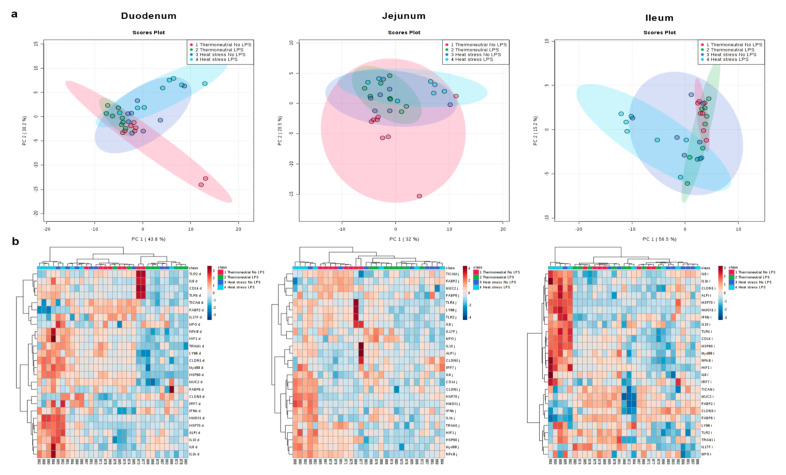
Principal component analysis (PCA) plots (**a**) and heat maps (**b**) of the expression of all measured genes in the duodenum, jejunum, and ileum of broiler either kept under thermoneutral conditions (10 h, 23 °C) or kept under heat stress conditions (10 h, 36 °C) receiving an oral dose of saline (0.9%; no LPS) or LPS (1 × 2 mg/kg b.w.). *n* = 8 animals/treatment.

Exploratory analysis (principal component analysis (PCA) and hierarchical clustering (heat map)) was performed on the expression of 26 genes related to LPS pathway, inflammatory response, gut health, and gut barrier for each intestinal part, respectively. Whereas the thermoneutral groups with or without LPS shared similar patterns all along the small intestine, heat stress seemed to have a significant impact on the gene expression profile (Figure 3). Separate clustering was clearly observed when both heat stress and oral LPS bolus were combined, especially in the duodenum and the ileum. Following exploratory analysis, differential expression analysis was done to determine the effects of the different treatments as well as the interaction between oral LPS administration and heat stress.

**Figure 4:**
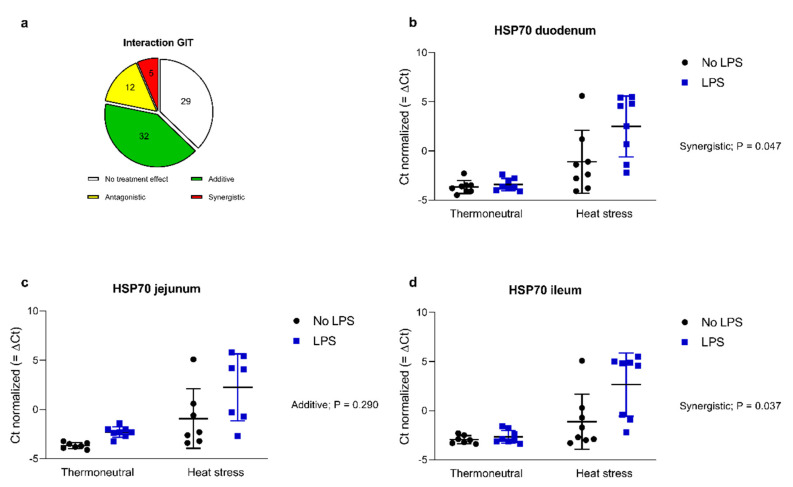
Summary of the number of interactions in the gastrointestinal tract (GIT) (**a**). Interactions of treatment factors on the delta Ct values of heat shock protein 70 (HSP70) in the duodenum (**b**), jejunum (**c**), and ileum (**d**) of broilers either kept under thermoneutral conditions (10 h, 23 °C) or kept under heat stress conditions (10 h, 36 °C) receiving an oral dose of saline (0.9%; no LPS) or LPS (1 × 2 mg/kg b.w.). *n* = 8 animals/treatment. Values present means with standard deviation.

When all results of the three intestinal parts were compiled (78 interactions studied), additive effects of the treatments (LPS × heat stress) were seen on 32 genes, whereas synergistic and antagonistic interactions were observed on 5 and 12 genes, respectively (Figure 4a). The strongest influence of heat stress combined with oral LPS administration was seen on the HSP70 gene in all parts of the gastrointestinal tract. A synergistic effect was observed for HSP70 in the duodenum as well as the ileum (Figure 4b,d), whereas there was an additive effect on HSP70 in the jejunum (Figure 4c). Heat stress alone significantly increased the expression of HSP70 all along the small intestine up to 6.7 times. LPS alone did not affect the mRNA level of HSP70, but dramatically potentiated the effect of heat stress in each intestinal segment with fold-increases ranging from 49 to 72 (Table 3).

The pro-inflammatory cytokine IL-1beta was another gene showing a synergistic interaction between LPS and heat stress in the duodenum and ileum (Table 3). Although not considered synergistic, the combination of LPS and heat stress led to an elevated expression of IL-6 in the duodenum (11.7-fold). Table 3 reports the most affected genes, and interestingly, the FABP2 gene encoding the intestinal-FABP protein showed a consistent decrease of its expression all along the small intestine. Furthermore, there was a significant decrease of TLR4 in the duodenum by fourfold. Expression of all of the genes can be found in Supplementary Tables S2–S4.

#### 2.2.4. Gene Expression Analyses of the Spleen and Liver

The expression of genes in the liver and the spleen was less affected by the treatments compared with the small intestine, with two genes significantly affected in the liver and four genes in the spleen. There was a synergistic effect on HSP70 and IL-1beta expression in the spleen, whereas there was an antagonistic effect on ceruloplasmin (CP) expression in the liver. Heat stress decreased the expression of CP by 5.2-fold in the liver, and TLR2 by 4.6-fold in the spleen (Table 4). Similar to the findings in the small intestine, heat stress alone increased the expression of HSP70 in the liver as well as in the spleen by 6.5-fold and 8-fold, respectively. Oral administration of LPS to heat stressed animals led to a 69.8-fold increase in the spleen. In addition, the combined treatment increased the expression of IL-1beta and IL-6 by 26.1-fold and 15-fold in the spleen, respectively. Expression of all genes can be found in Supplementary Tables S5 and S6.

#### 2.2.5. Serum 3-OH C14 and Acute Phase Protein Concentration

**Figure 5:**
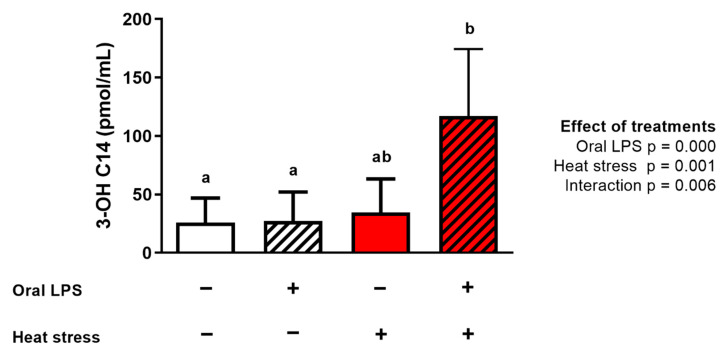
Mean serum 3-OH C14 concentration (pmol/mL) of broilers either kept under thermoneutral conditions (10 h, 23 °C) or kept under heat stress conditions (10 h, 36 °C) receiving an oral dose of saline (0.9%; no LPS) or LPS (1 × 2 mg/kg b.w.). *n* = 8 animals/treatment. Value are presented as mean with standard deviation. − indicates no treatment, + indicates treatment. ^ab^ Superscripts indicate significant difference (*p* < 0.05).

The 3-OH C14 concentration in the serum was significantly increased in heat stressed animals receiving LPS compared with both thermoneutral groups. The fold-change in comparison with the thermoneutral group receiving no LPS (4.5-fold) and thermoneutral group receiving LPS (4.3-fold) was similar (Figure 5). Overall, LPS administration, heat stress, as well as the interaction of both factors had a significant influence on the 3-OH C14 concentration. The effect of the treatments was synergistic. There was no effect of any treatment on the alpha-1-acid glycoprotein, ceruloplasmin, and serum amyloid A concentration in the serum (Table 5).

## 3. Discussion

Lipopolysaccharides are continuously released into the gut lumen. However, common scenarios in livestock production can challenge the well-balanced gut microbiome and lead to dysbiosis. During dysbiosis, shifts in the gut bacteria can result in increasing abundance of Gram-negative bacteria, especially Proteobacteria, and in excessive release of LPS [32]. In general, the presence of LPS in the intestine of healthy animals is considered harmless [32]. However, as soon as LPS translocate from the gut lumen into the circulation, they are extremely strong stimulators of inflammatory reactions even at low concentrations, and can induce sepsis in the host. The gut barrier plays a crucial role for endotoxin translocation. Different stressors can lead to a comprised gut barrier; that is, suboptimal diet composition [33], mycotoxins [34,35], heat stress [36], and weaning stress in pigs [37] and cattle [38]. It also needs to be considered that systemic LPS itself (e.g., resulting from any systemic Gram-negative infection) can impact the gut barrier owing to the stimulation and release of cytokines (e.g., TNF), leading to a vicious circle [39]. Overall, complex models combining more than one stressor are needed to assess the challenges broilers have to deal with in the livestock industry. Endotoxins need to be considered as one of the stressors resulting in decreased body weight gain, lower feed intake, as well as clinical or sub-clinical signs [40]. In the current study, we aimed at gaining a better understanding of the host response to LPS regarding (i) local response in the intestine and/or (ii) systemic response due to possible LPS translocation, as well as (iii) combined with other stressors such as heat stress. Additionally, we assessed the effect of three different LPS strains and commercial preparations to gain more insight into the LPS-activated pathways (MyD88-dependent and -independent), and the subsequent triggering of the expression of pro-inflammatory cytokines. As there is no commercial chicken macrophage cell line available, a murine macrophage cell line RAW 264.7 (commonly used to study the effect of LPS in vitro) was used to assess the inflammatory response of macrophages to different commercial LPS preparations. In total, all the tested LPS preparations induced the same strong inflammatory response in RAW cells, similarly affecting the same genes and pathways. Surprisingly, we did not observe any effect on toll-like receptor 4 (TLR4) expression at both time points, regardless of the LPS type used. Toll-like receptors belong to the pattern recognition receptor families. TLR4 is described as the major recognition receptor for LPS [41], which after interaction with extracellular CD14 and MD2 leads to the activation of two different pathways: MyD88-dependent and -independent pathway [42]. This eventually results in the transcription of several genes associated with the inflammatory response. Although we observed this strong upregulation of pro-inflammatory cytokines already after 4 h of LPS stimulation, this lack of effect on TLR4 could be the result of a negative feedback loop, as already described by Lichte et al. [43]. This could also account for the lack of or little effects (up to twofold increase) seen on the genes directly regulated and involved in the TLR4 signalling cascade, namely, CD14, TICAM1, MD2, and MyD88. We cannot exclude that LPS can be recognized through a TLR4-independent mechanism. Besides, residual protein impurities in the LPS preparations might affect the expression profile of macrophages as well. It is known that these impurities are able to activate another TLR, namely, TLR2. Li et al. [44] described that two lipoproteins constitute the major contaminant of LPS preparations and are responsible for the TLR2 activation. We further confirmed this observation in the present study with up-regulation of the expression of TLR2 by 2–3-fold after 4 h of LPS stimulation. In addition, a difference in IL-6 expression after 4 h and TNF concentration in the supernatant after 24 h stimulation was only observed for LPS from *E.coli* B4:111 with the IC purification compared with other LPS preparations tested. This might reflect the potency of different LPS preparations regarding the number of other components, for example, lipoproteins, nucleic acids, phospholipids, fragments of peptidoglycan, and polysaccharides, which strongly depends on the strain and purification method. Especially, the varying amount of lipoproteins in commercially available LPS prepared with PE seems to have a great influence on inflammatory response [45]. It needs to be considered that natural LPS occur as native endotoxins in its natural complexed cell membrane associated form. This is different from highly purified LPS, but these preparations help to understand and simulate the in vivo response of the host. This host response to LPS is overall less prominent in chickens than in other species, such as humans, calves, or rabbits [9]. Resistance of broiler chickens to LPS could partly be explained by the TLR4/MD2 complex in chicken, which has the unique property of lacking the functional-specific TRAM-TRIF signalling pathway (MyD88-independent) [10]. Therefore, LPS may only activate the MyD88-dependent signalling route, implying the differential in vivo response in chicken compared with other species. Although all LPS preparations induced a strong response of the pro-inflammatory genes, there was hardly a significant difference in the response between the different LPS preparations. However, there was a tendency that genes related to the MyD88-dependent pathway activation (e.g., IL-1beta, TNF-alpha, and IL-6) reacted stronger to stimulation with *E. coli* O55:B5 (PE) and *E. coli* O111:B4 (IC). Given *E. coli* O55:B5 is also commonly used in animal studies, and may allow better comparison with results from those studies, we decided to use this preparation of LPS derived from the phenol extraction for the in vivo phase in broiler chickens.

As discussed earlier, broilers are exposed to more than one stressor during livestock production. Heat stress is of significant importance in livestock industry; therefore, heat stress was included in the trial design as an additional stress factor to the oral LPS bolus. The present study is the first one to investigate the dynamic of an oral LPS bolus in the gut in broiler chickens, as well as the local and systemic effects of LPS alone or combined with a single heat stress exposure.

To evaluate the fate of the oral LPS bolus in the intestinal tract, the limulus amebocyte lysate (LAL) assay, a renowned assay to measure endotoxin activity [46], was used. In control animals, endotoxin activity increased from the upper parts of the intestine to the ileum, which is in accordance with data from a previous study by our group [47]. To the best of our knowledge, there are no other reports on the intestinal endotoxin concentration of healthy broilers, let alone studies investigating the endotoxin load in challenged broilers. As a consequence, it is difficult to verify whether our challenge model sufficiently mimics the increase of intestinal LPS during phases of gut dysfunction in broilers. In the current study, we observed that the LPS bolus led to an eightfold increase of the endotoxin activity in the jejunum in heat stressed animals, and a ninefold increase in the ileum in animals without heat stress. In ruminants, the endotoxin load can increase even up to 17-fold in the rumen and up to 7-fold in the cecum in dairy cows during a high-concentrate challenge model [44]. In our study, the basal endotoxin activity in the ileum was already very high compared with the proximal parts of the small intestine, independent of the treatment. This finding can be ascribed to the increase of gut microbiota all along the intestinal tract. Whereas in the ileum, the highest endotoxin activity was measured in the thermoneutral group receiving LPS, the pattern was different for the jejunum, where the combined treatment (LPS and heat stress) showed the strongest increase in endotoxin activity.

Gut motility might have been affected by heat stress, thereby transporting LPS slower in heat-stress animals. On one hand, a reduced gut passage rate might be a consequence of lower feed intake commonly described in heat stressed animals [36]. On the other hand, heat stress impairs intestinal blood flow, thus affecting gut motility as well [23]. However, the results should be handled with care as inter-individual differences were high.

Overall, the combined treatment had the biggest influence on the expression of measured genes in the intestine. Interestingly, expression of TLR4 was downregulated in the duodenum of animals receiving only the oral LPS bolus. This finding is very similar to our results in the murine macrophages, assuming a negative feedback loop. This downregulation of TLR4 was neither observed in any other part of the intestine of animals receiving the oral LPS bolus nor in any part of the intestine of heat stressed animals. This finding is in contrast with other studies, as they could see either an increase of the expression of TLR4 in the duodenum [48] or both in the jejunum and ileum [19] in heat stressed broilers. Increased TLR4 expression is often associated with gut barrier failure [49]. However, TLR4 expression was upregulated in the ileum when broilers received the oral LPS bolus combined with heat stress. It is known that TLR4 expression can be activated by heat shock proteins, and their expression was found to be strongly affected in the combined treatment. Heat shock proteins have a major role in the stress response and are considered as general markers for tissue injury [50,51]. Among them, HSP70 and HSP90 play an essential role in the regulation of protein homeostasis during physiological as well as pathological conditions [50,51]. Heat stress is known to affect the expression of HSP70 [19,48,52]; therefore, it was not surprising that HSP70 was also upregulated with heat stress alone (up to 6.7 fold-increase). However, the potentiation of this effect when the birds received the oral LPS bolus in combination with heat stress was unexpected. The increase of HSP70 expression was up to 70-fold in the duodenum, 60-fold in the jejunum, and 50-fold in the ileum. Likewise, spleen was mostly affected by the combined treatment, with a 70-fold increase of the HSP70 expression. The mechanism behind this highly selective and fast (within three hours) activation of HSP70 in the combination group remains unclear. However, HSP70 is known to serve as a danger signal or damage-associated molecular pattern molecule for the host immune system [53]. Interestingly, HSP70 and other HSPs bind a number of pathogen-associated molecules, such as LPS, and seem to also be involved in transfer of LPS to the TLR4/MD2 complex, as well as in LPS trafficking [54]. This topic deserves further investigation considering this strong and consistent effect on HSP70 all along the small intestine.

In addition to HSP70, other significant changes were observed in the gut, especially on the expression of MUC2 and FABP2. Both genes are discussed to be markers for gut health in livestock animals. MUC2 is important for mucin secretion and has protective properties to improve gut barrier function [55]. Downregulation of MUC2 might prevent the renewal of the mucous layer, thereby increasing the effect of other challenges and/or pathogen exposure. FABPs are intercellular chaperones and, among them, two are highly expressed in the small intestine (FABP2, FABP6) on the top of the villi. This is the initial site of destruction in several intestinal diseases. FABP2 is involved in fatty acid transport and lipogenesis, and plays an important role in the abdominal fat content of broilers [56,57]. FABP2 is further associated as a marker for gut barrier function in broiler chickens [33]. FABP6 is specific to the ileum, and is also involved in fatty acid transport and shows high affinity for bile acids. Expression of both genes, MUC2 and FABP2, was significantly downregulated in the jejunum of birds treated with LPS alone, but not in the duodenum and ileum. Similarly, heat stress reduced the expression of both genes in the jejunum. The strongest effect could again be seen with the combined treatment on both MUC2 and FABP2 with additive effects in all parts of the intestine. As expected, FABP6 expression was significantly affected in the ileum. To the best of our knowledge, there are no other studies available describing the effect of heat stress on MUC2, FABP2, or FABP6 gene in the intestine of broilers. Another gene that was affected by the combination of LPS and heat stress was ALPI. ALPI is an important enzyme produced in the gut, and plays an important role in intestinal homeostasis and interacts with the microbiome, diet, and the host [58]. This enzyme is important for the mucosal barrier, and is capable of detoxifying endotoxins [59]. Surprisingly, the highest upregulation of ALPI was observed in the ileum by fivefold in heat stressed animals receiving LPS, although the endotoxin load was highest in the jejunum compared with the other gut segments. Furthermore, a strong local inflammation response in the intestine was observed. The inflammation related genes IL-1beta and IL-6 were strongly upregulated in the duodenum as well as ileum in heat stressed animals receiving LPS. In addition, these two genes were also highly upregulated in the spleen, indicating a response to translocated LPS.

We could hardly see an effect of any treatment on the expression of the two tight junction proteins CLDN1 and CDLN3, which are associated with gut barrier function. This is in accordance with Santos et al. [52], reporting that one single day of heat stress was not enough to alter tight junction proteins, whereas after five days, this alteration occurred.

Thanks to their size, endotoxins are also discussed as a marker for paracellular gut permeability [60]. Therefore, information on endotoxin concentration in the blood can provide valuable information on the gut permeability. Our study is the first to measure blood 3-hydroxymyristic acid concentration (3-OH C14) via LC-MS/MS to assess endotoxin translocation from the gut into the blood in broiler. 3-OH C14 is a 3-hydroxy fatty acid present in the lipid A part of LPS, and thus correlates with the LPS concentration [61]. No increase of 3-OH C14 was observed in the blood of broilers that received the oral LPS bolus, confirming there was no influence on the gut barrier. Surprisingly, we could not find an increased 3-OH C14 concentration in the blood of broilers exposed to heat stress only. This is in contrast to studies in broilers [30] as well as pigs [25,28], where the authors reported an increase of blood endotoxin activity during heat stress. The severity, duration, and number of heat stress events seem to have a great impact on endotoxin translocation. The strong effects observed from the combined treatment on the expression of several genes in the gut might partly be explained by the fact that the serum 3-OH C14 concentration was increased by 4.5-fold in heat stressed animals receiving LPS. As a consequence, endotoxin circulating in the blood could have a direct effect in the intestine, affecting gene expression. Several studies have shown that *i.v.* and *i.p.* LPS application increase the expression of inflammatory genes and affect the gut barrier [40]. Therefore, increased endotoxin concentration in the blood might lead to a vicious circle, impairing the gut barrier function, and finally, more endotoxin can translocate to the blood. Furthermore, one study describes the effect of heat stress combined with subcutaneous injection of 100 µg/kg LPS derived from *Salmonella enterica typhimurium* [62]. In accordance with our study, the combined treatment had the most pronounced impact on gene expression.

Although 3-OH C14 concentration in the blood was significantly increased, there was hardly any effect of the combined treatment on the liver. Liver is known to be the most important organ to detoxify endotoxins once they have reached the liver [63,64]. Surprisingly, the expression of ceruloplasmin in the liver was even downregulated in heat stressed broilers. Therefore, the systemic response to the increased endotoxin concentration in the blood of heat stressed animals receiving the LPS bolus remains unclear.

## 4. Conclusions

The present study showed that short-term heat stress in combination with increased endotoxin activity in the gut, such as encountered during dysbiosis, results in additive and synergistic effects on the intestinal host response, and may impair broiler health. Disrupted gut health allowed translocation of LPS from the gut to the blood, and long-term exposure might further affect broiler health. The current study also confirmed that some parameters have the potential to be used as biomarkers for gut health (MUC2, FABP2) and stress response (HSP70, IL-1beta, IL-6) in broilers. Similarly, the measurement of 3-hydroxymyristic acid (3-OH C14) in the serum is a promising new approach to assess the translocation of LPS. These are important tools needed in the evaluation of strategies to counteract the negative effects of stressors occurring in broiler production.

## 5. Materials and Methods

### 5.1. In Vitro Trials

#### 5.1.1. Preparation of LPS Stock Solution for Stimulation of RAW Cells

LPS from three different strains (*E. coli* O55:B5, *E. coli* O111:B4; *S. enterica enteritidis*) either prepared with phenol extraction (PE) or with phenol extraction and additionally purified with ion exchange chromatography (IC) were purchased from Sigma Aldrich (St. Louis, MO, USA). LPS (5 mg) were weighed in a pyrogen-free glass tube (Charles River Laboratories, Wilmington, MA, USA) and 5 mL of sterile Dulbecco’s phosphate-buffered saline (DPBS; Gibco, Life Technologies, Carlsbad, CA, USA) was added. The solution was prepared at 500 rpm for 20 min, and aliquots were frozen in pyrogen-free glass tubes (Charles River, Wilmington, MA, USA) at −20 °C. Before adding LPS to the cells, the stock solution was thawed at 500 rpm at room temperature, and diluted in culture medium during continuous shaking at 500 rpm.

#### 5.1.2. Cell Culture—Murine Macrophage Cell Line RAW 264.7

Murine macrophage cell line (RAW 264.7) was purchased from a commercial culture collection (Cell lines service, Oppenheim, Germany). Cells were maintained in RPMI 1640 medium (Ready to use, Cell lines service, Eppelheim, Germany). Media contained L-glutamine as well as 10% fetal bovine serum. RAW 264.7 cells were cultivated at 37 °C in a 5% CO_2_ atmosphere (Galaxy 48 S, New Brunswick, Eppendorf, Hamburg, Germany). No antibiotics were added to the cell culture medium. Cells were subcultured two to three times a week until cells reached 70–80% confluence.

#### 5.1.3. Stimulation of RAW Cells with Different LPS Preparations

RAW 264.7 cells were seeded at a density of 2.5 × 10^5^ cells/well in 12-well plates (Eppendorf, Hamburg, Germany). Every well contained 2 mL medium and cells were cultivated for 24 h. Thereafter, cells were stimulated with LPS (100 ng/mL) from *E. coli* O55:B5 (PE and IC), *E. coli* O111:B4 (PE and IC), or *S. enterica enteritidis* (PE and IC) for 4 or 24 h. Treatments were performed in duplicate on two different plates for every independent experiment (*n* = 6 independent experiments). After 4 and 24 h of LPS stimulation, supernatants were either collected for immediate measurement of the nitrite concentration or stored at −20 °C for determination of the TNF-alpha concentration via Enzyme-Linked Immunosorbent Assay (ELISA). Cells were washed once with 1 mL DPBS per well, and 500 µL RNAlater^®^ stabilization solution (Ambion Inc., Austin, TX, USA) was added to each well. After storing the plates for 24 h at 4 °C, they were frozen at −80 °C until RNA isolation.

#### 5.1.4. Determination of Nitrite and TNF-Alpha Concentration in the Cell Culture Supernatant

The concentration of nitrite in the cell culture supernatant after 4 and 24 h of LPS stimulation was determined with a commercially available test kit (Griess Reagent System, Promega, Madison, WI, USA). Assay was performed according to the manufacturer’s manual. Absorbance was measured (within 30 min) at 540 nm and a reference wavelength of 630 nm (SynergyTM HT Multi-Mode Microplate Reader, Biotek Instruments, Winooski, VT, USA). The nitrite concentration was calculated from a nitrite standard curve. TNF-alpha concentration in the cell culture supernatant was measured with a commercially available mouse TNF-alpha Quantikine ELISA Kit (R&D Systems, Minneapolis, MN, USA). Assay was performed according to the manufacturer’s manual. Supernatants were either analysed undiluted or diluted at 1:10.

#### 5.1.5. RNA Extraction and Gene Expression

RNAlater^®^ was discarded from each well after removing the plate from −80 °C. Cells were washed with 500 µL DBPS, lysed, and homogenized. Total RNA was isolated using the RNeasy Mini Kit (Qiagen GmbH, Hilden, Germany), as described in the manufacturer’s instructions. Isolated RNA samples were shipped on dry ice to Qiagen GmbH (Hilden, Germany), where the measurement of concentration and quality of RNA (via Nano Drop spectrophotometer and RNA integrity number measurement), cDNA synthesis, and RT-qPCR was conducted. The threshold cycle (Ct) values for all genes were provided by Qiagen and used for data analysis. The 2^ΔΔCt^ method was used for determining the gene expression. First, the ΔCt (normalized Ct) value for each sample was calculated by subtracting the Ct value for the target gene from the mean Ct value of the two housekeeping genes. For each gene, the mean ΔCt for each experimental group was calculated, and subsequently used for statistical evaluation and expressing the fold change (= 2^ΔΔCt^ value). Cut-off values of <−2 or >2 were used to identify relevant changes in gene expression. The list of twelve selected genes and references is summarized in Supplementary Table S7. Glyceraldehyde 3-phosphate dehydrogenase (GAPDH) and ribosomal protein L32 (RPL32) were used as housekeeping genes for normalization.

### 5.2. In Vivo Trial

#### 5.2.1. Ethics Statement

The animal experiment was approved by the Austrian authorities at the Government of Lower Austria (LF1-TVG-39/031-2016) and the date of approval was 23 May 2016. All experimental procedures were performed under strict compliance with the European Guidelines for the Care and Use of Animals for Research Purpose (European Commission. 2010) and according to Austrian law.

#### 5.2.2. Birds and Feeding

Thirty-two one-day-old Ross 308 broilers were placed randomly into eight pens (four birds/pen). Pens (2.25 m²) contained clean wood shavings litter on paper towels, one drinker, and one round feeder. Animals were fed according to the Ross 308 recommendations. Starter diet was fed until day 14, thereafter, grower diet was fed until the end of the trial. Diet formulation and nutrient composition are summarized in Supplementary Tables S8 and S9. Body weight was recorded on days 1, 14, and 29.

#### 5.2.3. Heat Stress and Oral LPS Administration

After 28 d at thermoneutral conditions (according to the Ross 308 guideline), broilers were randomly assigned to four treatment groups: thermoneutral, thermoneutral + oral LPS, heat stress, heat stress + oral LPS. On day 29, half of the birds were kept under thermoneutral conditions (23 °C) and the other half of the birds were kept under heat stress conditions (36 °C) for ten hours. A data logger (Lascar Electronics Ltd. UK, Wiltshire, UK) was placed in every pen to control the temperature. After seven hours, LPS from *E. coli* O55:B5 (2 mg/kg b.w.; dissolved in a 0.9% saline solution in glass tubes) was orally administrated to half of the animals in the thermoneutral group and heat stress group. Animals without LPS treatment received the same amount of a 0.9% saline solution. Three hours after administration, all animals were sacrificed.

#### 5.2.4. Sampling

Birds were euthanized by an overdose of carbon dioxide. Blood was collected using endotoxin-free equipment during exsanguination. Serum was collected by centrifugation for 20 min at 2000× *g* at 4 °C. Serum was aliquoted into pyrogen-free reaction tubes (Sarstedt, Nümbrecht, Germany) and stored at −20 °C before further use. For gene expression analysis, a small piece of the duodenum, mid-jejunum, mid-ileum, liver, and spleen was collected and placed into RNAlater^®^. Tissue was kept overnight at 4 °C, and was stored thereafter at −80 °C until subsequent RNA isolation. Intestinal content was collected from the duodenum, jejunum, and ileum into pyrogen-free tubes (Sarstedt, Nümbrecht, Germany). Samples were stored at −20 °C before further analysis.

#### 5.2.5. Endotoxin Activity in the Intestine

Digesta samples (0.5 g) were homogenized with 5 mL endotoxin-free reagent water (Charles River, Wilmington, MA, USA). Diluted digesta was kept for 1 h at 300 rpm. Thereafter, samples were centrifuged for 10 min at 4500× *g* and 4 °C. Pellets were discarded, and supernatants were further diluted. Chromogenic limulus amebocyte lysate (LAL) assay (Charles River, Wilmington, MA, USA) was performed according to the manufacture’s manual. Instead of endotoxin-free reagent water, GlucaShield^®^ buffer (Associates of Cape Cod Europe GmbH, Mörfelden-Walldorf, Germany) was used to reconstitute LAL reagent to avoid interferences by (1→3)-ß-D-glucans. Spike controls were included.

#### 5.2.6. RNA Extraction and Gene Expression Analysis

Tissue samples (30–50 mg) were disrupted by bead-beating steps. Further procedure was done as described for RAW cells. The expression of selected genes was analysed via qPCR by Qiagen (Hilden, Germany). Data evaluation was done as described for RAW cells. Glyceraldehyde 3-phosphate dehydrogenase (GAPDH) and ribosomal protein L4 (RPL4) were used as housekeeping genes for normalization. A list of 28 (intestine) and 24 (liver, spleen) genes and references is summarized in Supplementary Table S10.

#### 5.2.7. Serum Endotoxin and Acute Phase Protein Concentration

The total endotoxin concentration (based on the 3-OH C14:0 concentration) in serum was assessed by HPLC-MS/MS according to Pais de Barros et al. [61] by EndoQuant (Dijon, France). Serum concentration of the acute phase proteins alpha 1-acid glycoprotein (AGP), ceruloplasmin (CER), and serum amyloid A (SAA) were measured using commercially available ELISA kits (Life diagnostics, West Chester, PA, USA). Assays were performed according to the manufacturer’s manual.

### 5.3. Statistics

Data analyses and graphs were done with GraphPad Prism Version 8 (GraphPad Software, San Diego, CA USA). All data were tested for normal distribution with the Kolmogorov–Smirnov test. If data were normally distributed, one-way analysis of variance (ANOVA) was performed with Bonferroni as the post-hoc test. If data were not normally distributed, Kruskal Wallis test was used as the non-parametric test with Dunn’s test as the post-hoc test. Differences were considered statistically significant when *p*-value was <0.05. Data from in vitro experiments were compared for 4 and 24 h, separately. Furthermore, data from the animal experiment were subjected to two-way ANOVA (factorial analysis). The latter included fixed effects of heat stress (thermoneutral, heat stress), oral LPS administration (yes, no), and their interaction. Two-way factorial ANOVA was carried out when at least one of the treatments was found to be significantly different from the control group to determine the type of interaction between treatments. Interaction was considered additive when *p*-value > 0.05 (i.e., no interaction), and synergistic or antagonistic when *p*-value < 0.05 (i.e., interaction). Principal component analysis (PCA) plots and heat maps were performed with the free webtool MetaboAnalyst 4.0 [65]. Normalized Ct values were used as input data.

## Figures and Tables

**Table 1:** Expression of selected genes in RAW cells stimulated with 100 ng/mL lipopolysaccharides (LPS) from two different *E. coli* strains (O55:B5, O111:B4) and one *S. enteritica enteritidis* strain either phenol extracted (PE) or phenol extracted and purified with ion-exchange chromatography (IC) for 4 h. *n* = 6. Values are fold changes expressed relative to untreated cells (Control).

	Control	*E. coli* O55:B5		*E. coli* O111:B4		*S. enteritica enteritidis*	
**Genes**		PE	IC	PE	IC	PE	IC
	*Genes involved in Toll-like receptor signalling cascade*						
**CD14**	1.0 ^a^	2.1 ^b^	2.0 ^b^	2.0 ^b^	2.1 ^b^	1.9 ^b^	2.0 ^b^
**MD2**	1.0	1.3	1.2	1.2	1.3	1.2	1.3
**TLR2**	1.0 ^a^	2.8 ^b^	2.6 ^b^	2.7 ^b^	2.7 ^b^	2.4 ^b^	2.5 ^b^
**TLR4**	1.0 ^a^	−2.5 ^b^	−2.6 ^b^	−2.5 ^b^	−3.1 ^b^	−2.3 ^b^	−2.4 ^b^
**MYD88**	1.0	1.8	1.8	1.6	1.6	1.5	1.7
**TICAM1**	1.0 ^a^	2.0 ^b^	1.7 ^ab^	2.0 ^b^	1.7 ^ab^	1.7 ^ab^	1.8 ^ab^
	*Genes triggered following Toll-like receptor activation*						
**IL-1beta**	1.0 ^a^	569 ^bc^	463 ^b^	502 ^bc^	652 ^c^	414 ^b^	434 ^b^
**TNF-alpha**	1.0 ^a^	10.2 ^bc^	9.7 ^bcd^	8.9 ^bd^	11.5 ^c^	7.8 ^bd^	8.5 ^bd^
**IL-6**	1.0 ^a^	854 ^b^	717 ^b^	635 ^ab^	915 ^b^	579 ^ab^	561 ^ab^
**iNOS**	1.0 ^a^	230 ^b^	216 ^b^	205 ^b^	175 ^b^	192 ^b^	222 ^b^
**IFNB1**	1.0 ^a^	279 ^b^	261 ^b^	222 ^ab^	298 ^b^	197 ^ab^	216 ^ab^
**IL-10**	1.0	1.9	2.3	1.7	2.7	1.4	1.2

^abcd^ Superscripts indicate significant difference (*p* < 0.05). CD14 = CD14 antigen, MD2 = Myeloid Differentiation factor 2, TLR2 = Toll-like receptor 2; TLR4 = Toll-like receptor 4; MYD88 = Myeloid differentiation primary response protein; TICAM1 = Toll-like receptor adaptor molecule 1; IL-1beta = Interleukin-1beta; TNF-alpha = Tumor necrosis factor alpha; IL-6 = Interleukin-6; iNOS = Inducible nitric oxide synthase; IFNB1 = Interferon beta 1 (IFNb1); IL-10 = Interleukin-10.

**Table 2:** Expression of selected genes in RAW cells stimulated for 24 h with 100 ng/mL LPS from two different *E. coli* strains (O55:B5, O111:B4) and one *S. enteritica enteritidis* strain either phenol extracted (PE) or purified with ion-exchange chromatography.

	Control	*E. coli* O55:B5		*E. coli* O111:B4		*S. enteritica enteritidis*	
**Genes**		PE	IC	PE	IC	PE	IC
*Genes involved in Toll-like receptor signalling cascade*							
**CD14**	1.0 ^a^	2.1 ^b^	2.1 ^b^	2.2 ^b^	2.3 ^b^	2.2 ^b^	2.1 ^b^
**MD2**	1.0	−1.0	−1.0	−1.0	1.2	1.0	1.0
**TLR2**	1.0	1.6	1.5	1.6	1.6	1.6	1.5
**TLR4**	1.0	−1.1	−1.2	−1.2	−1.1	−1.2	−1.2
**MYD88**	1.0	1.2	1.2	1.1	1.1	1.2	1.1
**TICAM1**	1.0	1.5	1.5	1.5	1.6	1.1	1.5
*Genes triggered following Toll-like receptor activation*							
**IL-1beta**	1.0 ^a^	81 ^b^	79 ^b^	101 ^b^	152 ^c^	102 ^b^	89 ^b^
**TNF-alpha**	1.0 ^a^	23 ^b^	22 ^b^	26 ^b^	26 ^b^	28 ^b^	23 ^b^
**IL-6**	1.0 ^a^	406 ^b^	369 ^b^	499 ^b^	811 ^b^	510 ^b^	421 ^b^
**iNOS**	1.0 ^a^	36 ^b^	43 ^b^	38 ^b^	52 ^b^	51 ^b^	42 ^b^
**IFNB1**	1.0	−1.3	−1.1	−1.1	−1.3	1.1	1.1
**IL-10**	1.0 ^a^	−4.5 ^b^	−3.5 ^b^	−4.7 ^b^	−2.8 ^b^	−3.1 ^b^	−2.8 ^b^

^ab^ Superscripts indicate significant difference (*p* < 0.05). CD14 = CD14 antigen, MD2 = Myeloid Differentiation factor 2, TLR2 = Toll-like receptor 2; TLR4 = Toll-like receptor 4; MYD88 = Myeloid differentiation primary response protein; TICAM1 = Toll-like receptor adaptor molecule 1; IL-1beta = Interleukin-1beta; TNF-alpha = Tumor necrosis factor alpha; IL-6 = Interleukin-6; iNOS = Inducible nitric oxide synthase; IFNB1 = Interferon beta 1 (IFNb1); IL-10 = Interleukin-10.

**Table 3:** Fold change of the expression of the six most affected genes in the duodenum, jejunum, and ileum of broilers either kept under thermoneutral conditions (10 h, 23 °C) or kept under heat stress conditions (10 h, 36 °C) receiving an oral dose of saline (0.9%; no LPS) or LPS (1 × 2 mg/kg b.w.). *n* = 8 animals/treatment. Thermoneutral group was set to 1.0.

Six Most Affected Genes.						
**Duodenum**						
**Treatments**	**HSP70**	**IL-6**	**IL-1beta**	**TLR2**	**FABP2**	**TLR4**
Thermoneutral	1.0 ^a^	1.0 ^a^	1.0 ^a^	1.0 ^a^	1.0 ^a^	1.0 ^a^
Thermoneutral + LPS	1.2 ^a^	2.7 ^ab^	−1.2 ^a^	−5.3 ^b^	−1.9 ^a^	−4.2 ^b^
Heat stress	5.9 ^a^	3.3 ^ab^	1.6 ^a^	−1.3 ^ab^	−1.6 ^a^	1.5 ^a^
Heat stress + LPS	71.8 ^b^	11.7 ^b^	7.0 ^b^	−6.0 ^bc^	−5.8 ^b^	2.7 ^a^
**Interactions**						
Type	synergistic	additive	synergistic	additive	additive	antagonistic
*p*-value	0.047	0.763	0.023	0.878	0.258	0.006
**Jejunum**						
**Treatments**	**HSP70**	**ALPI**	**FABP2**	**HSP60**	**MUC2**	**CLDN1**
Thermoneutral	1.0 ^a^	1.0 ^a^	1.0 ^a^	1.0 ^a^	1.0 ^a^	1.0 ^a^
Thermoneutral + LPS	2.5 ^a^	3.0 ^ab^	−3.5 ^b^	−2.0 ^ab^	−3.1 ^b^	1.2 ^a^
Heat stress	6.7 ^ab^	2.4 ^ab^	−1.9 ^a^	−3.6 ^b^	−2.7 ^b^	2.1 ^ab^
Heat stress + LPS	59.5 ^b^	5.6 ^b^	−4.2 ^ab^	1.3 ^a^	−3.2 ^b^	3.2 ^b^
**Interactions**						
Type	additive	additive	additive	antagonistic	additive	additive
*p*-value	0.292	0.701	0.404	0.001	0.047	0.592
**Ileum**						
**Treatments**	**HSP70**	**FABP6**	**FABP2**	**IL-6**	**ALPI**	**IL-1beta**
Thermoneutral	1.0 ^a^	1.0 ^a^	1.0 ^a^	1.0 ^a^	1.0 ^a^	1.0 ^a^
Thermoneutral + LPS	1.2 ^a^	−1.7 ^a^	−2.9 ^ab^	−1.1 ^a^	−1.3 ^a^	1.1 ^a^
Heat stress	3.6 ^b^	−2.7 ^a^	−1.5 ^a^	4.3 ^b^	1.6 ^ab^	1.4 ^a^
Heat stress + LPS	49.4 ^b^	−9.2 ^b^	−7.7 ^b^	7.0 ^b^	4.6 ^b^	4.0 ^b^
Interactions *						
Type	synergistic	additive	additive	additive	additive	synergistic
*p*-value	0.037	0.245	0.447	0.323	0.056	0.037

^abc^ Superscripts indicate significant difference (*p* < 0.05). * *p* < 0.05, interaction between LPS and heat stress is considered as synergistic or antagonistic. *p* > 0.05, interaction between LPS and heat stress is considered as additive. HSP70 = Heat shock protein 70; IL-6 = Interleukin-6; IL-1beta = Interleukin-1beta; TLR2 = Toll-like receptor 2; FABP2 = Fatty acid binding protein 2; TLR4 = Toll-like receptor 4; ALPI = Intestinal alkaline phosphatase; HSP60 = Heat shock protein 60; MUC2 = Mucin 2; CLDN1 = Claudin 1; FABP6 = Fatty acid binding protein 6; IL-6 = Interkeukin-6.

**Table 4:** Fold-change of the expression of affected genes in the liver and spleen of broilers either kept under thermoneutral conditions (10 h, 23 °C) or kept under heat stress conditions (10 h, 36 °C) receiving an oral dose of saline (0.9%; no LPS) or LPS (1 × 2 mg/kg b.w.). *n* = 8 animals/treatment. Thermoneutral group was set to 1.0.

	Liver		Spleen			
	HSP70	CP	HSP70	IL-1beta	IL-6	TLR2
**Treatments**						
Thermoneutral	1.0 ^ab^	1.0 ^a^	1.0 ^a^	1.0 ^a^	1.0 ^a^	1.0 ^a^
Thermoneutral + LPS	−3.9 ^a^	−1.9 ^ab^	1.1 ^a^	1.3 ^a^	2.0 ^ab^	−1.0 ^a^
Heat stress	6.5 ^b^	−5.2 ^b^	8.0 ^b^	2.0 ^a^	−1.2 ^a^	−4.6 ^b^
Heat stress + LPS	4.5 ^ab^	−1.1 ^ab^	69.8 ^c^	26.1 ^b^	15.0 ^b^	−8.5 ^b^
Interactions *						
Type	additive	antagonistic	synergistic	synergistic	additive	additive
*p*-value	0.558	0.003	0.033	0.006	0.058	0.422

^abc^ Superscripts indicate significant difference (*p* < 0.05). * *p* < 0.05, interaction between LPS and heat stress is considered as synergistic or antagonistic. *p* >0.05, interaction between LPS and heat stress is considered as additive. HSP70 = Heat shock protein 70; CP = Ceruloplasmin; IL-1beta = Interleukin-1beta; IL-6 = Interleukin-6; TLR2 = Toll-like receptor 2.

**Table 5:** Mean blood AGP (µg/mL), CER (µg/mL), and SAA (ng/mL) concentration of broilers either kept under thermoneutral conditions (10 h, 23 °C) or kept under heat stress conditions (10 h, 36 °C) receiving an oral dose of saline (0.9%; no LPS) or LPS (1 × 2 mg/kg b.w.) (*n* = 8 animals/treatment).

	Acute Phase Protein (Mean ± SD)		
Treatments	Alpha 1 Acid Glycoprotein (AGP) (µg/mL)	Ceruloplasmin (CER) (µg/mL)	Serum Amyloid A (SAA) (ng/mL)
Thermoneutral	1647 ± 1111	409 ± 97	353 ± 528
Thermoneutral + LPS	912 ± 459	399 ± 47	944 ± 1680
Heat stress	1142 ± 741	415 ± 54	217 ± 260
Heat stress + LPS	1296 ± 1068	393 ± 103	1085 ± 1662
*p*-Value	0.551	0.968	0.278

## Supplementary Materials

The following are available online at https://www.mdpi.com/2072-6651/12/10/622/s1, Table S1 Mean body weight ± SD of broiler without any treatment on day 1 (*n* = 16 animals/treatment) and 14 (*n* = 16 animals/treatment). Table S2 Fold-change of the expression of all measured genes in the duodenum. Table S3 Fold-change of the expression of all measured genes in the jejunum. Table S4 Fold-change of the expression of all measured genes in the ileum. Table S5 Fold-change of the expression of all measured genes in the liver. Table S6 Fold-change of the expression of all measured genes in the spleen. Table S7 Selected genes and references analysed in RAW cells. Table S8 Diet formulation of the starter and grower diet. Table S9 Nutrient composition of the starter and grower diet. Table S10 Selected genes and references for gene expression analysis of the duodenum, jejunum, ileum, liver, and spleen of the in vivo trial with broiler.
