# Supplementary material for: Endotoxin Translocation and Gut Inflammation Are Increased in Broiler Chickens Receiving an Oral Lipopolysaccharide (LPS) Bolus during Heat Stress
Source: Toxins (Basel). 2020 Sep 29;12(10):622. doi: 10.3390/toxins12100622 (PMC7601408; doi:10.3390/toxins12100622)
Supplement: Supplementary file 1 [file toxins-12-00622-s001.pdf]

Supplementary files: Endotoxin Translocation and Gut Inflammation are Increased in Broiler Chickens Receiving an Oral Lipopolysaccharide (LPS) Bolus during Heat Stress

Nicole Reisinger*, Caroline Emsenhuber, Barbara Doupovec, Elisabeth Mayer, Gerd Schatzmayr, Veronika Nagl, and Bertrand Grenier

Table S1. Mean body weight ± SD of broiler without any treatment on day 1 (n = 16 animals/treatment) and 14 (n = 16 animals/treatment). Mean body weight ± SD of broiler at day 29 before applying thermoneutral conditions (23 °C) or kept under heat stress conditions (36 °C) receiving an oral dose of 0.9% saline (No LPS) or LPS (2 mg/kg b.w.) (n = 8 animals/treatment).

Treatment	BW (g/bird)		
	**Day 1**	**Day 14**	**Day 29***
**Thermoneutral**	43 ± 0.4	365 ± 57	1226 ± 197
**Thermoneutral + LPS**			1208 ± 255
**Heat stress**	43 ± 0.0	345 ± 87	1199 ± 289
**Heat stress + LPS**			1262 ± 202
***p*-value**	n.a.	0.4605	0.9556

*Measured before heat stress and LPS bolus. n.a. = not assessed.

Table S2. Fold change of the expression of all measured genes in the duodenum.

Duodenum									
	LPS Pathway								
**Treatments**	**CD14**	**TLR2**	**TLR4**	**MD2**	**MYD88**	**TICAM**	**TRAM**	**IRF7**	**NF-kB**
Thermoneutral	1.0 [ab]	1.0 [a]	1.0 [a]	1.0 [ab]	1.0 [ab]	1.0	1.0	1.0 [a]	1.0
Thermoneutral + LPS	−2.4 [a]	−5.3 [b]	−4.2 [b]	−1.6 [a]	−1.6 [b]	−1.5	−1.3	−1.0 [a]	−1.8
Heat stress	−1.1 [ab]	−1.3 [ab]	1.5 [a]	1.6 [ab]	1.4 [a]	−1.2	1.1	1.2 [a]	1.1
Heat stress + LPS	2.4 [b]	−6.0 [bc]	2.7 [a]	2.3 [b]	2.6 [a]	−1.4	1.1	2.0 [b]	1.8
**Interactions***									
**Type**	antagonistic	additive	antagonistic	additive	antagonistic	additive	additive	additive	antagonistic
**P-values**	0.018	0.878	0.006	0.153	0.012	0.508	0.132	0.100	0.020
	**Stress Response**		**Inflammation Response**						
**Treatments**	**HSP60**	**HSP70**	**IL-1β**	**IL-6**	**IL-8**	**IL-10**	**IL-17F**	**IFNB**	
Thermoneutral	1.0 [ab]	1.0 [a]	1.0 [a]	1.0 [a]	1.0 [ab]	1.0 [a]	1.0 [ab]	1.0	
Thermoneutral + LPS	−2.6 [a]	1.2 [a]	−1.2 [a]	2.7 [ab]	−2.8 [b]	1.3 [ab]	−1.5 [ab]	1.3	
Heat stress	−1.2 [ab]	5.9 [a]	1.6 [a]	3.3 [ab]	1.9 [a]	2.6 [ab]	2.2 [a]	1.5	
Heat stress + LPS	1.5 [b]	71.8 [b]	7.0 [b]	11.7 [b]	3.8 [a]	4.3 [b]	−2.4 [b]	1.7	
**Interactions***									
**Type**	antagonistic	synergistic	synergistic	additive	additive	additive	additive	no effect	
**P-values**	0.008	0.047	0.023	0.763	0.071	0.794	0.110	0.802	
	**Oxidative Stress**		**Gut Permeability**			**Gut Health**			
**Treatments**	**HIF1-α**	**HMOX**	**CLDN1**	**CLDN3**	**MUC2**	**ALPI**	**FABP2**	**FABP6**	**MPO**
Thermoneutral	1.0 [ab]	1.0	1.0	1.0	1.0	1.0 [a]	1.0 [a]	1.0	1.0
Thermoneutral + LPS	−1.4 [a]	1.0	−1.5	1.3	−1.9	1.0 [a]	−1.9 [a]	−1.1	−1.0
Heat stress	1.2 [ab]	1.3	1.8	1.7	1.2	2.2 [ab]	−1.6 [a]	−1.1	1.4
Heat stress + LPS	2.5 [b]	3.8	2.5	1.5	1.4	3.6 [b]	−5.8 [b]	1.8	−1.2
**Interactions***									
**Type**	antagonistic	additive	additive	additive	additive	additive	additive	no effect	no effect

**P-values**	0.008	0.125	0.236	0.277	0.074	0.360	0.258	0.096	0.505

^{abc} Superscripts indicate significant difference ($p < 0.05$). * $p < 0.05$ interaction between LPS and heat stress is considered as synergistic or antagonistic. $p > 0.05$, interaction between LPS and heat stress is considered as additive. no effect = No significant effect of any treatment on parameter.

Table S3. Fold change of the expression of all measured genes in the jejunum.

**Jejunum**									
**LPS Pathway**									
**Treatments**	**CD14**	**TLR2**	**TLR4**	**MD2**	**MYD88**	**TICAM**	**TRAM**	**IRF7**	**NF-kB**
Thermoneutral	1.0	1.0	1.0	1.0 ^{a}	1.0	1.0	1.0	1.0	1.0 ^{a}
Thermoneutral + LPS	1.5	−3.8	−1.3	−3.7 ^{ab}	−1.6	−1.3	−1.2	1.1	−2.0 ^{b}
Heat stress	2.0	−2.4	−1.3	−1.6 ^{b}	−1.1	−1.1	−1.0	1.2	−1.2 ^{a}
Heat stress + LPS	3.2	−5.2	2.0	−1.1 ^{a}	1.8	−1.1	−1.1	1.5	1.2 ^{a}
**Interactions***									
**Type**	no effect	additive	no effect	antagonistic	antagonistic	no effect	no effect	no effect	antagonistic
**P-values**	0.915	0.513	0.082	0.006	0.013	0.209	0.170	0.635	0.001
	**Stress Response**		**Inflammation Response**						
**Treatments**	**HSP60**	**HSP70**	**IL-1β**	**IL-6**	**IL-8**	**IL-10**	**IL-17F**	**IFNB**	
Thermoneutral	1.0 ^{a}	1.0 ^{a}	1.0	1.0	1.0	1.0	1.0	1.0	
Thermoneutral + LPS	−2.0 ^{ab}	2.5 ^{a}	2.0	2.4	−3.6	2.5	1.3	1.2	
Heat stress	−3.6 ^{b}	6.7 ^{ab}	1.2	2.8	−2.9	1.8	−1.3	1.3	
Heat stress + LPS	1.3 ^{a}	59.5 ^{b}	2.9	3.4	−3.0	1.8	−1.1	2.2	
**Interactions***									
**Type**	antagonistic	additive	additive	additive	no effect	no effect	no effect	no effect	
***p* -values**	0.001	0.292	0.774	0.360	0.104	0.194	0.858	0.581	
	**Oxidative Stress**		**Gut Permeability**		**Gut Health**				
**Treatments**	**HIF1-α**	**HMOX**	**CLDN1**	**CLDN3**	**MUC2**	**ALPI**	**FABP2**	**FABP6**	**MPO**
Thermoneutral	1.0	1.0	1.0 ^{a}	1.0	1.0 ^{a}	1.0 ^{a}	1.0 ^{a}	1.0	1.0
Thermoneutral + LPS	−1.2	−1.2	1.2 ^{a}	2.0	−3.1 ^{b}	3.0 ^{ab}	−3.5 ^{b}	−1.8	2.4
Heat stress	−1.0	1.1	2.1 ^{ab}	2.5	−2.7 ^{b}	2.4 ^{ab}	−1.9 ^{a}	−1.4	−1.4

Heat stress + LPS	1.3	3.8	3.2^{b}	2.1	−3.2^{b}	5.6^{b}	−4.2^{ab}	−4.4	−1.3
**Interactions***									
**Type**	no effect	additive	additive	no effect	additive	additive	additive	no effect	no effect
***p* -values**	0.119	0.053	0.592	0.076	0.047	0.701	0.404	0.682	0.269

^{ab} Superscripts indicate significant difference ($p < 0.05$). * $p < 0.05$ interaction between LPS and heat stress is considered as synergistic or antagonistic. $p > 0.05$, interaction between LPS and heat stress is considered as additive. no effect = No significant effect of any treatment on parameter.

Table S4. Fold change of the expression of all measured genes in the ileum.

.	**Ileum**								
	**LPS Pathway**								
**Treatments**	**CD14**	**TLR2**	**TLR4**	**MD2**	**MYD88**	**TICAM**	**TRAM**	**IRF7**	**NF-kB**
Thermoneutral	1.0	1.0	1.0^{ab}	1.0	1.0	1.0	1.0	1.0	1.0
Thermoneutral + LPS	−1.2	−1.1	−1.1^{a}	−1.1	−1.2	−1.3	−1.1	−1.3	−1.1
Heat stress	1.4	−1.5	1.4^{ab}	−1.2	−1.1	−1.2	1.1	1.1	−1.0
Heat stress + LPS	2.4	−2.3	2.6^{b}	1.2	1.2	−1.4	1.0	1.8	1.4
**Interactions***									
**Type**	no effect	no effect	no effect	no effect	no effect	additive	no effect	antagonistic	no effect
***p*-values**	0.305	0.722	0.150	0.255	0.048	0.708	0.841	0.005	0.157
	**Stress Response**		**Inflammation Response**						
**Treatments**	**HSP60**	**HSP70**	**IL-1β**	**IL-6**	**IL-8**	**IL-10**	**IL-17F**	**IFNB**	
Thermoneutral	1.0^{ab}	1.0^{a}	1.0^{a}	1.0^{a}	1.0	1.0	1.0	1.0	
Thermoneutral + LPS	−1.4^{a}	1.2^{a}	1.1^{a}	−1.1^{a}	−1.2	1.1	1.1	−1.2	
Heat stress	−1.0^{ab}	3.6^{b}	1.4^{a}	4.3^{b}	−1.4	1.2	−3.8	1.3	
Heat stress + LPS	2.8^{b}	49.4^{b}	4.0^{b}	7.0^{b}	1.4	2.3	−2.6	1.3	
**Interactions***									
**Type**	antagonistic	synergistic	synergistic	additive	no effect	no effect	additive	additive	
***p*-values**	0.035	0.037	0.037	0.323	0.181	0.219	0.733	0.642	
	**Oxidative Stress**		**Gut Permeability**			**Gut Health**			
	**HIF1-α**	**HMOX**	**CLDN1**	**CLDN3**	**MUC2**	**ALPI**	**FABP2**	**FABP6**	**MPO**

Thermoneutral	1.0	1.0	1.0	1.0	1.0	1.0 ^{a}	1.0 ^{a}	1.0 ^{a}	1.0
Thermoneutral + LPS	1.0	−1.3	1.1	−1.5	−2.1	−1.3 ^{a}	−2.9 ^{ab}	−1.7 ^{a}	−1.4
Heat stress	−1.0	1.3	1.6	1.3	−1.9	1.6 ^{ab}	−1.5 ^{a}	−2.7 ^{a}	−1.6
Heat stress + LPS	1.6	6.1	2.0	−1.5	−2.6	4.6 ^{b}	−7.7 ^{b}	−9.2 ^{b}	−1.8
**Interactions***									
**Type**	no effect	synergistic	additive	additive	no effect	additive	additive	additive	no effect
***p*-values**	0.115	0.0421	0.677	0.674	0.598	0.056	0.447	0.245	0.707

^{ab} Superscripts indicate significant difference ($p < 0.05$). * $p < 0.05$ interaction between LPS and heat stress is considered as synergistic or antagonistic. $p > 0.05$, interaction between LPS and heat stress is considered as additive. no effect = No significant effect of any treatment on parameter.

Table S5. Fold change of the expression of all measured genes in the liver.

					**Liver**					
					**LPS Pathway**					
**Treatments**	**CD14**	**TLR2**	**TLR4**	**MD2**	**MYD88**	**TICAM**	**TRAM**	**IRF7**	**NF-kB**	
Thermoneutral	1.0	1.0	1.0	1.0	1.0	1.0	1.0	1.0	1.0	
Thermoneutral + LPS	1.1	1.5	−1.9	−1.4	−1.3	1.0	1.0	−1.2	−1.1	
Heat stress	−1.4	−3.6	−2.0	−2.0	1.0	−1.0	−1.4	−1.1	−1.5	
Heat stress + LPS	1.4	−1.1	1.0	1.7	1.2	1.0	1.0	1.8	1.8	
**Interactions***										
**Type**	no effect	no effect	antagonistic	antagonistic	no effect	no effect	additive	no effect	antagonistic	
***p*-values**	0.272	0.469	0.035	0.013	0.139	0.801	0.057	0.112	0.042	
	**Stress Response**			**Inflammation Response**				**Acute Phase Response**		
**Treatments**	**HSP60**	**HSP70**	**IL-1β**	**IL-6**	**IL-8**	**IFNB**	**LTF**	**OGCHI**	**CP**	**SAAL1**
Thermoneutral	1.0	1.0 ^{ab}	1.0	n.a	1.0	1.0	1.0	1.0	1.0 ^{a}	1.0
Thermoneutral + LPS	−1.1	−3.9 ^{a}	1.2	n.a.	1.2	−1.1	−1.3	−1.8	−1.9 ^{ab}	−1.2
Heat stress	−1.3	6.5 ^{b}	1.0	n.a.	−1.9	1.5	−1.4	−1.6	−5.2 ^{b}	−1.8
Heat stress + LPS	1.5	4.5 ^{ab}	2.9	n.a.	4.7	−1.1	1.1	−1.2	−1.1 ^{ab}	1.6
**Interactions***										
**Type**	no effect	additive	no effect	n.a.	no effect	no effect	no effect	antagonistic	antagonistic	antagonistic

***p*-values**	0.145	0.558	0.389	n.a.	0.150	0.329	0.055	0.023	0.003	0.015

^{ab} Superscripts indicate significant difference ($p < 0.05$). * $p < 0.05$ interaction between LPS and heat stress is considered as synergistic or antagonistic. $p > 0.05$, interaction between LPS and heat stress is considered as additive. no effect = No significant effect of any treatment on parameter. n.a. = not assessed.

Table S6. Fold change of the expression of all measured genes in the spleen.

**Spleen**										
	**LPS Pathway**									
**Treatments**	**CD14**	**TLR2**	**TLR4**	**MD2**	**MYD88**	**TICAM**	**TRAM**	**IRF7**	**NF-kB**	
Thermoneutral	1.0	1.0 ^{a}	1.0	1.0	1.0	1.0	1.0	1.0	1.0	
Thermoneutral + LPS	−1.3	−1.0 ^{a}	−1.0	1.0	1.1	−1.1	−1.0	1.2	1.1	
Heat stress	−2.1	−4.6 ^{b}	−1.7	−1.2	−1.6	−1.4	−1.4	−1.6	−1.5	
Heat stress + LPS	−2.1	−8.5 ^{b}	−1.2	1.3	1.5	−1.5	−1.3	1.8	1.4	
**Interactions***										
**Type**	additive	additive	no effect	no effect	antagonistic	no effect	additive	antagonistic	additive	
***p*-values**	0.574	0.422	0.487	0.261	0.026	0.392	0.799	0.013	0.648	
	**Stress Response**		**Inflammation Response**					**Acute Phase Response**		
**Treatments**	**HSP60**	**HSP70**	**IL-1β**	**IL-6**	**IL-8**	**IFNB**	**LTF**	**OGCHI**	**CP**	**SAAL1**
Thermoneutral	1.0 ^{ab}	1.0 ^{a}	1.0 ^{a}	1.0 ^{a}	1.0	1.0	1.0	1.0	1.0	1.0
Thermoneutral + LPS	−1.2 ^{ab}	1.1 ^{a}	1.3 ^{a}	2.0 ^{ab}	1.2	1.1	−1.2	1.3	−1.0	−1.1
Heat stress	−2.0 ^{a}	8.0 ^{b}	2.0 ^{a}	−1.2 ^{a}	−2.6	1.3	−2.0	1.9	1.1	−1.2
Heat stress + LPS	1.4 ^{b}	69.8 ^{c}	26.1 ^{b}	15.0 ^{b}	3.2	−1.8	1.4	6.5	1.9	1.5
**Interactions***										
**Type**	antagonistic	synergistic	synergistic	additive	antagonistic	no effect	no effect	no effect	no effect	no effect
***p*-values**	0.000	0.033	0.006	0.058	0.025	0.184	0.075	0.504	0.395	0.073

^{ab} Superscripts indicate significant difference ($p < 0.05$). * $p < 0.05$ interaction between LPS and heat stress is considered as synergistic or antagonistic. $p > 0.05$, interaction between LPS and heat stress is considered as additive. no effect = No significant effect of any treatment on parameter.

Table S7. Selected genes and references analysed in RAW cells.

Selected Genes (LPS Pathway, Inflammation)	Reference
CD14 antigen (CD14)	NM_009841
Lymphocyte antigen 96 (Ly69) = Myeloid Differentiation factor 2 (MD2)	NM_001159711
Interleukin 1 beta (IL1b)	NM_008361
Tumor necrosis factor alpha (TNF-alpha)	NM_013693
Interleukin 6 (IL6)	NM_031168
Nitric oxide synthase 2, inducible (iNOS)	NM_010927
Interferon beta 1 (IFNb1)	NM_010510
Toll-like receptor 2 (TLR2)	NM_011905
Toll-like receptor 4 (TLR4)	NM_021297
Myeloid differentiation primary response gene 88 (MyD88)	NM_010851
Toll-like receptor adaptor molecule 1 (TICAM1)	NM_174989
Interleukin 10 (IL10)	NM_010548
**House Keeping Genes**	
Ribosomal protein L32 (RPL32)	NM_172086
Glyceraldehyde-3-phosphate dehydrogenase (GAPDH)	NM_008084

Table S8. Diet formulation of the starter and grower diet.

Ingredients.	Diets	
**Raw Materials, %**	**Starter Diet (Day 1–14)**	**Grower Diet (Day 15–29)**
Corn	53.63	60.79
Soya	31.34	25.08
Full Fat Soya	6.00	6.00
Calcium carbonate	1.50	0.88
Mono calcium phosphate	1.78	1.58
Fat powder	1.93	1.82
Soya oil	0.80	1.17
Potatoe Protein	0.70	0.60
Sodium bicarbonate	0.11	0
L-Lysine	0.31	0.23
DL-Methionine	0.24	0.19
Sodium chloride	0.33	0.32
Magnesium oxyd	0.15	0.15
L-Threonine	0.07	0.04
Cholinchloride	0.13	0.17
Vitamin and trace element premix	1.00	1.00

Table S9. Nutrient composition of the starter and grower diet.

Nutrients (g/kg DM)	Diets	
	**Starter Diet (Day 1–14)**	**Grower Diet (Day 15–29)**
Crude protein	216	191
Crude fat	65	68
Fibre	29	27
Raw ash	67	55

Calcium	13	10
Phosphorus	8	7
Sodium	2	2
Magnesium	3	3
Potassium	10	9
Chlorin	2	2
Starch	357	399
Sugar	53	48
Metabolic energy, MJ	13	13
Lysin	13	11
Methionin	5	4
Methionin + Cystin	8	7
Tryptophan	2	2
Threonin		8
Vitamin A (I.E)	10 000	9 500
Vitamin D (I.E)	5 000	4275
Vitamin E mg	100	70
Vitamin C mg	100	0
Vitamin K mg	3	3
Vitamin B1 mg	3	3
Vitamin B2 mg	8	8
Vitamin B6 mg	6	6
Vitamin B12 μg	40	38

Table S10. Selected genes and references for gene expression analysis of the duodenum, jejunum, ileum, liver and spleen of the in vivo trial with broiler.

Selected Genes (LPS Pathway, Inflammation)	Reference
Toll-like receptor 2 (TLR2)	NM_001161650
Toll-like receptor 4 (TLR4)	NM_001030693
CD14 antigen (CD14)	NM_001139478
Lymphocyte antigen 96 (LY96) = Myeloid Differentiation factor 2 (MD2)	XM_001232092
Myeloid differentiation primary response protein (MYD88)	NM_001030962
Interferon-beta (IFNW1)	NM_001024836
Interleukin 6 (IL6)	NM_204628
Interleukin 1 beta (IL1B)	NM_204524
Interleukin 8 (IL8)	NM_205018
Interferon regulatory factor 3 (IRF7)	NM_205372
Toll-like receptor adaptor molecule 1 (TICAM)	NM_001081506
Translocating chain-associated membrane protein 1 (TRAM1)	NM_204400
Nuclear factor kappa-B, subunit 1 (NFkB1)	NM_205134
Heat shock 70kDa protein 2 (HSPA2)	NM_001006685
Heat shock protein family D (Hsp60) member 1 (HSPD1)	NM_001012916
Interleukin 17A (IL17)	NM_204460
Interleukin 10 (IL10)	NM_001004414
Myoloperoxidase (MPO)	XM_415716
Heme oxygenase 1 (HMOX1)	NM_205344
Hypoxia-inducible factor 1 alpha (HIF1A)	NM_204297

**Selected Genes (Gut Health, Gut Barrier)***	
Claudin 1 (CLDN1)	NM_001013611
Claudin 3 (CLDN3)	NM_204202
Mucin 2 (MUC2)	XM_421035
Intestinal fatty acid binding protein 2 (FABP2)	NM_001007923
Intestinal fatty acid binding protein 6 (FABP6)	XM_414486
Intestinal alkaline phosphatase (ALPI)	XM_422743
**Acute Phase Proteins****	
Transferrin (Ovotransferrin) (TF)	NM_205304
Orosomucoid 1 (Ovoglycoprotein) (ORM1)	NM_204541
Ceruloplasmin (CP)	XM_001235148
Serum amyloid like A (SAAL1)	XM_003641328
**House Keeping Genes**	
Ribosomal protein L4 (RPL4)	NM_001007479
Glyceraldehyde-3-phosphate dehydrogenase (GAPDH)	NM_204305

*Measured only in the duodenum, jejunum and ileum, **Measured only in the liver and spleen.
